# Transcriptomic profile of tobacco in response to *Phytophthora nicotianae* infection

**DOI:** 10.1038/s41598-017-00481-5

**Published:** 2017-03-24

**Authors:** Jian-Kang Yang, Zhi-Jun Tong, Dun-Huang Fang, Xue-Jun Chen, Ke-Qin Zhang, Bing-Guang Xiao

**Affiliations:** 1 Key Laboratory of Tobacco Biotechnological Breeding, Yunnan Academy of Tobacco Agricultural Sciences, Kunming, 650021 China; 2 State Key Laboratory for Conservation and Utilization of Bio-Resource in Yunnan, Yunnan University, Kunming, 650021 China; 3 Department of Biochemistry and Molecular Biology, Dali University, Dali, 671000 China

## Abstract

Black shank, caused by *Phytophthora nicotianae* (*P. nicotianae*), is a serious disease of cultivated tobacco (*Nicotiana tabacum*) worldwide. The interactions between tobacco and *P. nicotianae* are complex and the outcomes of the interactions depend on the tobacco genotype, *P. nicotianae* strain, and environmental conditions. In this study, we used RNA-sequencing (RNA-Seq) to investigate and compare transcriptional changes in the stems of tobacco upon inoculation with *P. nicotianae* strain race 0. We used two tobacco varieties: RBST (named from resistance to black shank and tobacco mosaic virus), which was resistant to the *P. nicotianae* strain race 0, and Honghuadajinyuan (HD), which was susceptible to *P. nicotianae* race 0. Samples were collected 12 and 72-hour post inoculation (hpi). Analysis of differentially expressed genes (DEGs) and significantly enriched GO terms indicated that several basic defense mechanisms were suppressed in both varieties, which included response to wounding (GO: 0009611), and defense response to fungus (GO: 0050832). We also found some genes that may especially be related to mechanisms of resistance in RBST, such as the one encoding a chitinase. These results will provide a valuable resource for understanding the interactions between *P. nicotianae* and tobacco plants.

## Introduction

Black shank, caused by *Phytophthora nicotianae* (*P. nicotianae*), is among the most widespread and damaging diseases of cultivated tobacco (*Nicotiana tabacum*) worldwide^1^. The pathogen infects roots, stems, and leaves at any stage of plant growth, resulting in root rot, stem lesions, leaf necrosis, and plant death. Disease damage is increased rapidly under conditions of high temperature^2, 3^.

Management of tobacco black shank has relied upon an integrated approach that includes crop rotation, fungicide applications, and the use of resistant cultivars. The most cost-efficient method of managing the disease is the use of resistant cultivars^4^. Described physiological races of *P. nicotianae* include races 0, 1, 2, and 3, with races 0 and 1 currently having the highest prevalence^5^. Previous studies using tobacco cultivars with moderate or high levels of resistance have found that race 0 has higher pathogenic and ecologic fitness levels than race 1, suggesting that more attention should be paid to race 0^6^. Complete resistance to race 0, the most common race of *P. nicotianae*, is found in *Nicotiana plumbaginifolia* ^7^. *N. plumbaginifolia* is a wild tobacco species that is generally used as a parent in genetic breeding programs. Such cultivars with this type of resistance are also immune to race 0.

The knowledge of resistant mechanisms of tobacco to *P. nicotianae* is limited. The mechanism of resistance is complex and may be related to an array of physiological processes, such as affecting infection effectiveness, influencing recognition between pathogens and their hosts, blocking hyphal expansion, and extracellular accumulation of a cytotoxic activity^8–10^. Understanding the response of tobacco to *P. nicotianae* is important for developing strategies for disease control. As complicated as the mechanisms of disease resistance in plants can be, it is generally accepted that global investigation of gene expression profiles during disease infections could help to identify key components of resistance pathways^11^. So far, there has not been any report on the transcriptome of tobacco in response to *P. nicotianae* infection. Two tobacco varieties, Honghuadajinyuan (HD) and RBST, show different resistant levels to *P. nicotianae*. Here we performed RNA sequencing analysis of gene expression profiles in both HD and RBST tobacco stems, which were infected with *P. nicotianae* at two time points: 12 h and 72 h post inoculation. By comparing the gene expression patterns of HD and RBST, we found that some plant defense genes were differentially expressed between the two varieties. The acquired transcriptome data provide an invaluable resource for understanding the response/resistance to *P. nicotianae* infection in tobacco.

## Results

### Symptoms of black shank on tobacco

Symptoms were not apparent on HD and RBST at 12 h after inoculation. At 72 h, the inoculation site on the HD stem turned from brown to black; the darkening extended up and down for several centimeters, and the plant was wilted. The inoculation site on the RBST stem also turned from brown to black at 72 h, but the darkening did not spread, and the plant grew well.

### Annotation of unigenes in tobacco

**Figure 1:**
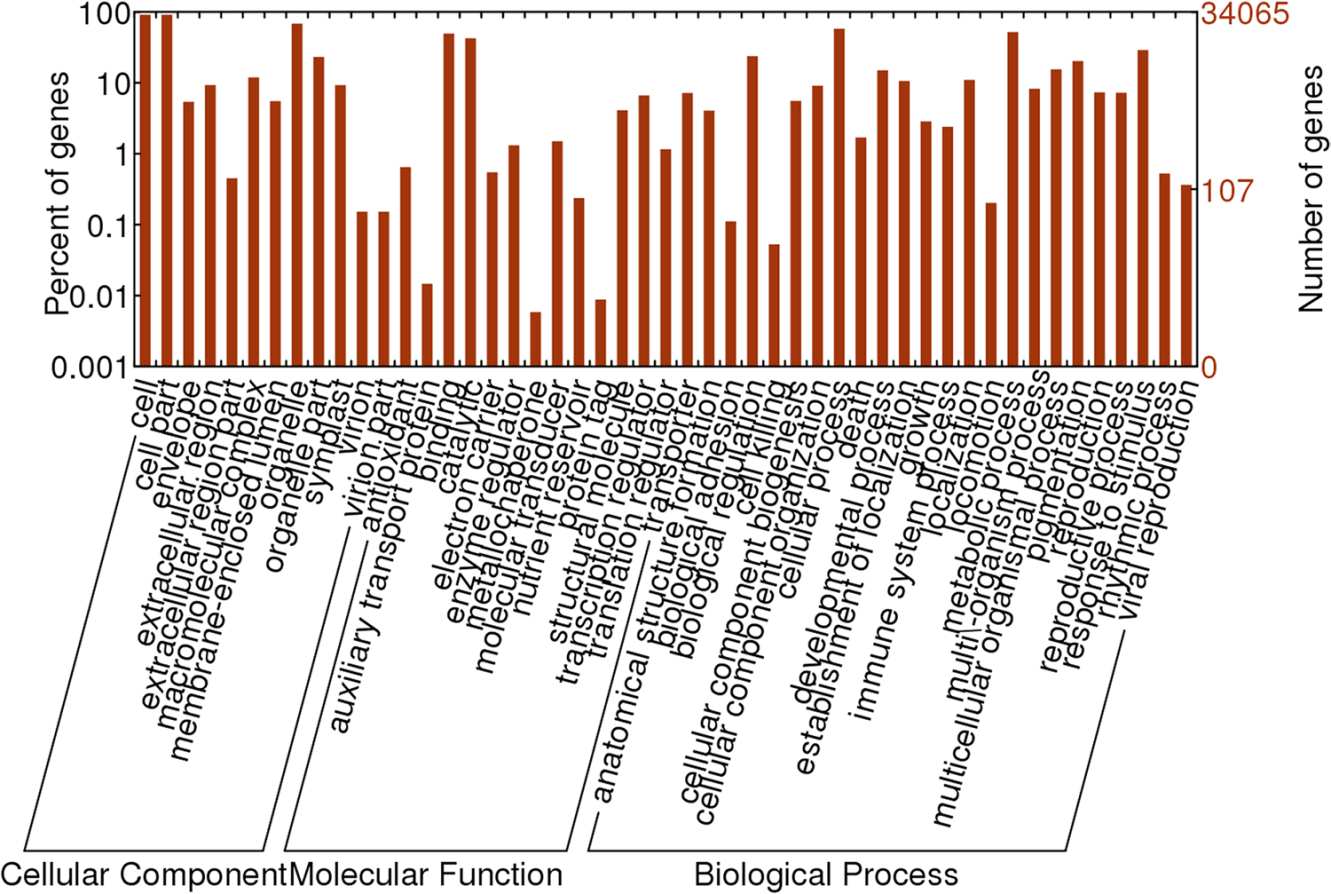
GO classification of annotated transcriptomic genes. Three levels (Biological Process, Molecular Function and Cellular Component) were demonstrated. The 34,065 transcripts were distributed among 49 GO terms.

We first defined the transcriptome by RNA-Seq. A total of 946,093,872 clean reads (141.92 Gb) were generated by Illumina RNA-Seq deep sequencing (Table 1). The reads of all samples (inoculated RBST, non-inoculated RBST, inoculated HD, and noninoculated HD) were pooled to generate a de novo assembly of transcriptomes. Initial assembly using Trinity yielded 316,948 contigs with an N50 value of 732 bp. Further prediction of open reading frames using TransDecoder obtained 49,935 unigenes, with an N50 value of 2,112 bp and a GC content of 40.05%. The N50 value was similar to those reported in other *de novo* assembly studies of tobacco^12^. We performed gene function annotation analysis of the 49,935 unigenes. The transcripts were aligned to the NCBI Non-Redundant Protein Database (NR database) using the blastp program (E-value 1e^−5^), allowing the annotation of 45,234 unigenes based on 32,161 NCBI non-redundant proteins. There were 39,127 unigenes that were represented by nearly full-length NR proteins, having > 80% alignment coverage. The Gene Ontology database was then utilized to retrieve the GO identifiers for each of the annotated genes. In total, 34,065 unigenes mapped to GO terms. We identified 49 main GO terms in three levels (23 GO terms in Biological Process, 14 GO terms in Molecular Function and 12 GO terms in Cellular Component) (Fig. 1).

**Table 1:** Statistics of Illumina sequencing data.

Samples	Raw reads	Clean reads	Clean bases	Q20 (%)	Q30 (%)	Notes
HD_72 h_1	55,333,426	52,820,790	7.92 G	96.48	91.8	replicate 1
HD_72 h_2	54,001,208	51,490,186	7.72 G	96.48	91.77	replicate 2
HD_72 h_3	53,280,954	51,467,222	7.72 G	96.24	91.34	replicate 3
HD_12 h_1	62,883,914	60,129,216	9.02 G	96.25	91.56	replicate 1
HD_12 h_2	58,867,280	56,178,894	8.43 G	96.41	91.81	replicate 2
HD_12 h_3	51,914,688	49,516,308	7.43 G	96.45	91.92	replicate 3
HD_ck_1	58,054,930	55,815,774	8.37 G	96.41	91.71	replicate 1
HD_ck_2	58,003,858	55,727,534	8.36 G	96.25	91.41	replicate 2
HD_ck_3	43,483,010	41,760,824	6.26 G	96.24	91.44	replicate 3
RBST_72 h_1	51,844,458	48,777,122	7.32 G	95.77	90.46	replicate 1
RBST_72 h_2	59,056,054	55,180,180	8.28 G	95.85	90.58	replicate 2
RBST_72 h_3	54,507,452	51,240,810	7.69 G	95.51	89.99	replicate 3
RBST_12 h_1	50,379,538	47,397,668	7.11 G	95.07	89.23	replicate 1
RBST_12 h_2	54,390,186	51,261,418	7.69 G	95.25	89.61	replicate 2
RBST_12 h_3	49,216,216	46,394,664	6.96 G	95.23	89.55	replicate 3
RBST_ck_1	64,574,816	61,315,592	9.2 G	96.56	91.92	replicate 1
RBST_ck_2	59,395,738	57,072,576	8.56 G	96.5	91.85	replicate 2
RBST_ck_3	54,730,068	52,547,094	7.88 G	96.42	91.7	replicate 3
Total	993,917,794	946,093,872	141.92 G	96.08	91.09

### The amount of *P. nicotianae* in the infected samples

It is unknown whether there were different trends for the change in the number of the pathogen during its infection of different tobaccos, we analyzed the read numbers that were derived from *P. nicotianae*. There were 1.9% and 2.3% reads were derived from *P. nicotianae* for HD in 12 h and 72 h. There were 1.4% and 2.2% reads were derived from *P. nicotianae* for RBST in 12 h and 72 h. The late stage of the infection had more reads of *P. nicotianae* than that at the early stage. The trends were similar for the two tobaccos. However, the susceptible variety HD had more reads of *P. nicotianae* than that the resistant variety RBST had at the early stage of infection.

### Differentially expressed genes for each variety at two time points after infection

The gene expression profiles of healthy HD and RBST stems were used as controls. All genes and their gene expression data are listed in Supplementary Table S1 and Supplementary Table S2. If the gene expression in infected stems recorded a 4-fold (or more) difference relative to the control (*P* < 0.05), this gene was regarded as the differentially expressed gene (DEG).

**Figure 2:**
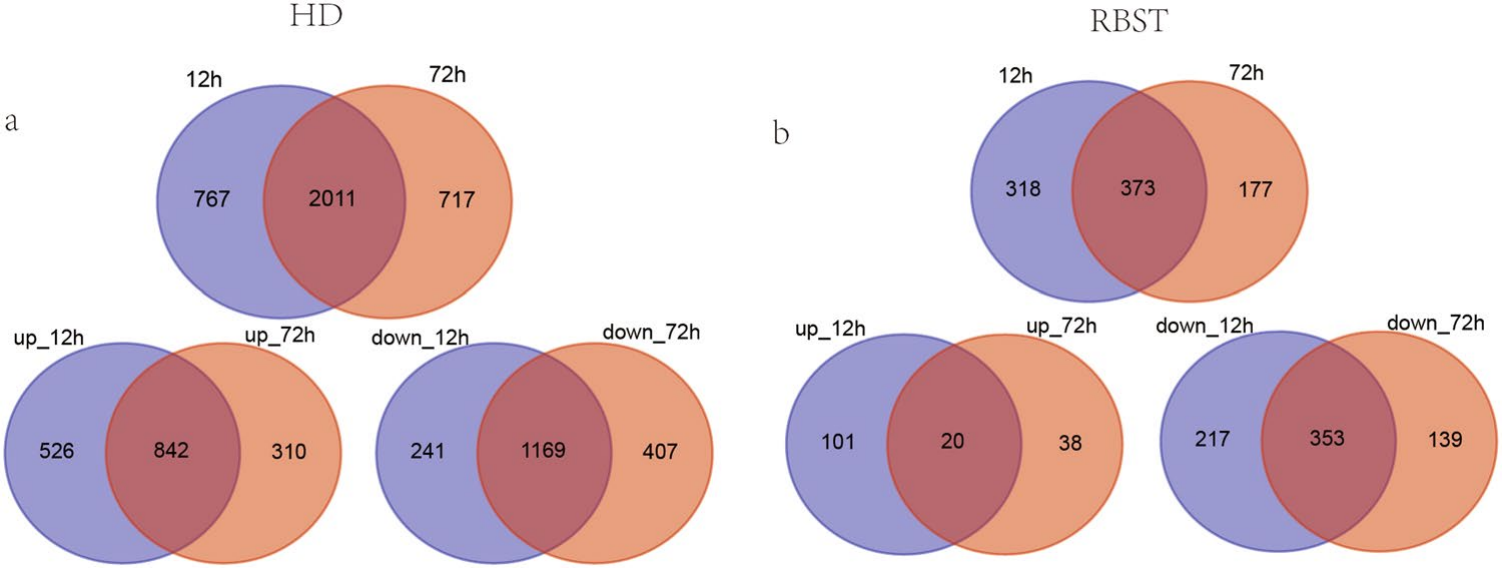
Summary for the differentially expressed genes. Venn diagram showing the number of specific and common differentially expressed genes. (**a**) Differentially expressed genes of HD between 12 h and 72 h. (**b**) Differentially expressed genes of RBST between 12 h and 72 h.

For HD, there were 2,778 DEGs at 12 h, and 2,728 DEGs at 72 h after inoculation. The number of upregulated DEGs at 12 h after inoculation was 1,368, and 1,152 at 72 h (Fig. 2a). For RBST, the number of DEGs decreased from 691 at 12 h after inoculation to 550 at 72 h after inoculation. The number of upregulated DEGs was lower than that of downregulated DEGs at 12 h (121 vs. 570 genes) and 72 h (58 vs. 492 genes) after inoculation (Fig. 2b).

As shown in Fig. 2, a Venn diagram was generated from the DEG lists at 12 h, and 72 h after inoculation to identify shared members. There were 2,011 differentially expressed genes at all time points in HD. The number of upregulated and downregulated DEGs were 842 and 1,169, respectively. For RBST, there were 373 DEGs that could be identified at all-time points. The number of upregulated and downregulated DEGs were 20 and 353, respectively.

As shown in Fig. 2, for HD, there were 767 DEGs that were only detected at 12 h and 717 DEGs that were only detected at 72 h. The numbers of upregulated DEGs were 526 at 12 h and 310 at 72 h, respectively. The numbers of downregulated DEGs were 241 at 12 h and 407 at 72 h, respectively. For RBST, there were 318 DEGs that only discovered in 12 h and 177 DEGs that were only discovered in 72 h. The numbers of upregulated DEGs were 101 at 12 h and 38 at 72 h, respectively. The numbers of downregulated DEGs were 217 at 12 h and 139 at 72 h, respectively.

### Shared differentially expressed genes during *P. nicotianae* infection

**Figure 3:**
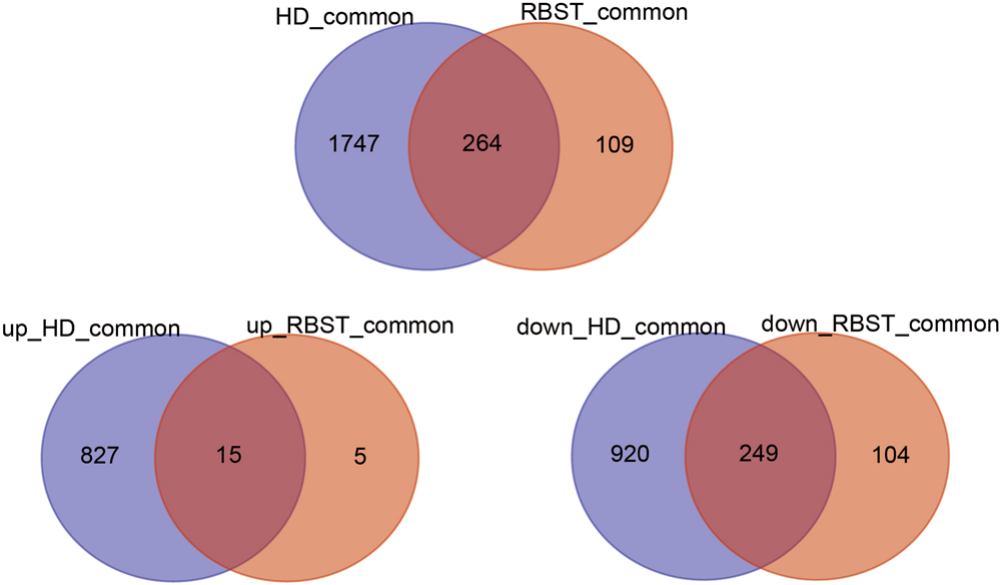
Venn diagram showing the commonalities and differences in two lists of shared differentially expressed genes. Shared differentially expressed genes are those differentially expressed at both 12 h and 72 h in HD or RBST.

The above-mentioned two lists of shared DEGs were further analyzed for commonalities and differences. As shown in the Venn diagram in Fig. 3, there were 264 common DEGs in two lists of DEGs. We were especially interested in the features of the 15-upregulated common DEGs (Supplementary Table S3). One gene, TR75953_c0_g12, was predicted to encode dehydration-responsive element-binding protein. This gene was involved in abscisic acid (ABA) -independent defense against pathogens in *Arabidopsis thaliana* and tobacco^13, 14^. Another gene, TR30085_c0_g2, encode a zinc finger protein. It participates in regulation of transcription. It is possible that it is important for the response to pathogens^15^. The functions of other DEGs were not clear.

Additionally, different numbers of unique DEGs were found for each variety: 1,747 unique DEGs in HD, and 109 unique DEGs in RBST. Especially for the resistant variety RBST, there were five upregulated DEGs (Supplementary Table S4). One gene, TR75736_c2_g1, which encodes a chitinase, is usually involved in the response to fungal pathogens^16^.

### Gene ontology (GO) enrichment analysis of differentially expressed genes

Within 49,935 identified unigenes, the protein products of 34,065 unigenes were annotated with at least one GO term. Using these 34,065 unigenes as references, 526 unique upregulated DEGs in HD_12 h, and 310 unique upregulated DEGs in HD_72 h, were enriched in 53 and 41 GO terms, respectively. These 53 GO terms were involved in “plastid part (GO: 0044435)” and “chloroplast thylakoid (GO: 0009534)” (Supplementary Table S5). These 41 GO terms were involved in “biological regulation (GO: 0065007)” and “gibberellin biosynthetic process (GO: 0009686)” (Supplementary Table S6). Similarly, 101 unique upregulated DEGs in RBST_12 h, and 38 unique upregulated DEGs in RBST_72 h, were enriched in 6 and 6 GO terms (Supplementary Table S7). These GO terms at 12 h were involved in “cell junction assembly (GO: 0034329) and “secondary cell wall (GO: 0009531)”. The GO terms at 72 h were involved in “defense response, incompatible interaction (GO: 0009814)” and “innate immune response (GO: 0045087)”.

There were 264 common DEGs between HD and RBST including 15 upregulated and 249 downregulated DEGs. For upregulated DEGs, they were enriched in seven GO terms, such as “organelle (GO: 0043226)” (Supplementary Table S8). For downregulated DEGs, 119 GO terms were identified such as “response to wounding (GO: 0009611)” and “defense response to fungus (GO: 0050832)” (Supplementary Table S9).

Only five upregulated DEGs were specifically identified and two GO terms were specifically enriched in RBST. They were “exochitinase activity (GO: 0035885)” and “endochitinase activity (GO: 0008843)”.

### Quantitative RT-PCR (qRT-PCR) validation of differentially expressed genes

A subset of 10 genes, which responded to *P. nicotianae* infection, were selected for quantitative real-time PCR (qRT-PCR) analyses (Supplementary Fig. S1). qRT-PCR analyses showed the trends of expression to be consistent with those found by RNA-Seq.

## Discussion

Black shank, as one of the most important plant diseases, causes crop losses worldwide^17^. In order to identify tobacco genes involved in broad-spectrum resistance to tobacco black shank, a previous study using suppression subtractive hybridization (SSH) was performed in *Nicotiana megalosiphon* to identify differentially expressed cDNAs, and 48 differentially expressed genes were discovered^18^. In this study, we used next-generation sequencing approaches to investigate the gene expression changes associated with the characteristic disease development process induced in tobacco plants by *P. nicotianae*. The results will assist in the discovery and annotation of important genes in plant defense response, physiology and metabolism. RNA-Seq analysis identified a total of 49,935 unigenes in tobacco. GO analysis showed that 34,065 of the unigenes could be grouped into 49 known GO terms. Our analyses of GO terms generally represented the main biological GO classification and ensured the integrity of the downstream functional analyses of the candidate genes.

It is worth mentioning that various unique GO terms were enriched at different time points. For example, the cellular component GO terms plastid part (GO: 0044435) and chloroplast thylakoid (GO: 0009534) were only enriched in HD after 12 h of infection. At 72 h, GO terms biological regulation (GO: 0065007) and gibberellin biosynthetic process (GO: 0009686) were enriched. Close examination of these GO terms may provide molecular insight into the mechanism of response to pathogens at different times after infection. The chloroplast is a photosynthesis-related organelle. Photosynthesis has been reported to modulate plant defense responses induced by pathogen infection^19^. At 72 h, some new biological processes were enhanced, such as gibberellin biosynthetic process. Previous studies have proven that the function of plant hormone signal transduction plays an important role in the defense response^20^. It is interesting that increasing the content of gibberellin in *N. benthamiana* plants could enhance the susceptibility of these plants to infectious pathogens^21^. One gene, TR75953_c0_g12, was upregulated in the HD both at 12 h and 72 h. This gene was involved in abscisic acid (ABA)-independent defense against pathogens in *Arabidopsis thaliana* and tobacco^13, 14^. ABA has been considered a negative regulator of disease resistance. These may be some reasons why HD is susceptible to *P. nicotiana*. Similarly, some special GO terms for RBST were identified at different time points. Cell junction assembly (GO: 0034329) was enriched at 12 h, and innate immune response (GO: 0045087) was enriched at 72 h. Our results further revealed that several genes related to innate immunity, e.g., *ADR1* and *RPM1* ^22, 23^, were also induced during systemic symptom development. These results indicated that the two varieties had different mechanisms to respond to *P. nicotianae* infection at different time points.

Our differentially expressed gene (DEG) analysis provided further insight into the molecular mechanism for common biological processes between resistant and susceptible tobaccos after *P. nicotianae* infection. Analysis of significantly enriched GO terms indicated that several biological processes are common between the two varieties. Most of the upregulated DEGs are enriched in cellular component GO terms, which were related to organelle construction. On the other hand, the downregulated DEGs belong to biological processes: response to wounding (GO: 0009611) etc. These downregulated biological processes belonged to defense-related pathways. And these pathways played important roles in defense response^24^. Those down-regulated genes in these pathways were likely to decrease basal defense response and reduce disease resistance to *P. nicotianae* infection in both resistant and susceptible tobaccos. It is well known that plant pathogens have evolved to secrete effectors, which can manipulate the host immune system and suppress host defense^25^. Previous papers have evidenced that some resistance (R) genes can be activated by specific effectors, which is shown by the accumulation of higher levels of R genes transcripts. Therefore, we paid attention to those putative R genes that could possibly be induced by *P. nicotianae* infection of the two varieties. Among these genes, TR29096_c1_g6 and TR29096_c0_g1 were commonly up-regulated in HD and RBST. These R proteins directly or indirectly detect bacterial or fungal effectors, which will activate downstream signaling and lead to pathogen resistance^26^.

The variety RBST has high levels of resistance to *P. nicotianae* race 0. However, why it could avoid the development of symptoms was not clear. In this study, we found some genes and biological processes that may be related to this resistant mechanism. Gene TR75736_c2_g1, which encodes chitinase, participated in exochitinase activity (GO: 0035885) and endochitinase activity (GO: 0008843). The expression level of this gene was 10 times higher than that of the control. *In vitro* assays in rice indicated that the chitinase showed antifungal activity with a clear inhibitory effect on the growth of the pathogen *Rhizoctonia solani* ^27^. The gene (TR24473_c0_g2) encoding plasma membrane protein, was upregulated in RBST. The plasma membrane is involved in important cellular process that determines signal response of plant to pathogen such as hypersensitive response^28^. There was no overlap between the 48 genes identified by SSH study^18^ and genes identified by our RNA-seq study. This may due to the different species used in these studies. The SSH study was performed in *Nicotiana megalosiphon*.

Genetic resources for resistance to black shank in the varieties of tobacco currently in use for production are mainly derived from Florida 301^29^ and *N. plumbaginifolia* ^30^. The varieties currently used to produce commercial flue-cured tobacco are not completely immune to black shank, and the effects can still be seen when pathogen levels exceed a critical threshold. Therefore, to reduce losses in tobacco production, it will be essential to perform more studies to identify genes and pathways involved in resistance to black shank.

In conclusion, the gene expression patterns of HD and RBST after *P. nicotianae* infection provide a solid foundation for future studies of the molecular mechanisms underlying the response of tobacco to black shank.

## Materials and Methods

### Inoculation of tobacco with *P. nicotianae*

The resistant tobacco breeding line RBST and the susceptible tobacco cultivar Honghuadajinyuan (HD) were used in the experiments. The two varieties of *N. tabacum* were developed by our institution. The detailed development processes of the two varieties had been described in the Supplement Materials and Methods. A field-isolate of *P. nicotianae* race 0 was used for all inoculations throughout this study. The inoculum and the protocol for inoculation under greenhouse conditions were prepared as described^31^. The infected stem was harvested at two time points, 12 and 72 h post inoculation.

### Sample collection and library preparation

All three biological replicate samples were used for RNA extraction. Total RNA was extracted from inoculated as well as noninoculated plants using TRIzol reagent (Invitrogen Corp., Carlsbad, CA). RNA purifications were performed using an RNeasy Mini Kit (Qiagen, Chatsworth, CA). Library preparation was carried according to the Illumina Hiseq RNA sample preparation kit (Illumina, San Diego, CA). All original data were deposited in the NCBI Sequence Read Archive database (accession number: SRP074868).

### De novo assembly and annotation of transcriptomes

Raw data (raw reads) in fastq format were first processed through the NGS QC Toolkit^32^. In this step, clean data (clean reads) were obtained by removing reads containing adapter, reads containing ploy-N, and low quality reads from raw data. At the same time, Q20, Q30, and GC-content of the clean data were calculated. All downstream analyses were based on clean data. The black shank resistance gene in RBST was derived from *N. plumbaginifolia* ^30^. The three varieties that had reference genomes do not have the introgression segment with resistance gene conferring resistance to *P. nicotianae* race 0^33^. So *de novo* assembled transcriptome was necessary. The transcriptome was *de novo* assembled using Trinity with the default parameters^34^. The candidates with the most probable longest ORF were generated from the Trinity assembly result using TransDecoder (https://transdecoder.github.io/). Protein sequences corresponding to the coding sequences of unigenes were obtained and searched against the NCBI non-redundant (nr) database, using blastp with a cut-off E-value of 10^−5^. Gene Ontology (GO) annotation was based on the Gene Ontology Database of Arabidopsis thaliana^35^. WEGO software was used to perform functional annotation analyses at three gene ontology levels (Biological Process, Molecular Function and Cellular Component)^36^.

### Detection of differentially expressed genes

RSEM^37^, an accurate transcript quantification tool for RNA-Seq data, was utilized to quantify transcripts. Briefly, clean data were mapped back onto the assembled transcriptome. The read count for each gene was obtained from the mapping results. Differential expression analysis between inoculated and noninoculated plants was performed using the DESeq^38^ and EdgeR^39^ package. The resulting *P* values were adjusted using the Benjamini and Hochberg’s approach for controlling the false discovery rate (FDR)^40^. The overlap between the sets of genes found by DESeq and edgeR with FDR < 0.05 and the absolute value of Log2 (Ratio) ≥ 2 were assigned as differentially expressed genes (DEGs). Venn Diagrams were drawn with VENNY (http://bioinfogp.cnb.csic.es/tools/venny).

### Gene ontology enrichment analyses

We identified GO terms at three levels (biological process, molecular function and cellular component). GO terms enriched in genes from the differentiation analyses were identified with KOBAS software^41^. GO terms with a false discovery rate (FDR)^42^ of less than 0.05 were considered over-represented.

### Quantitative real-time PCR (qRT-PCR) validation

To validate the DEG results, 10 DEGs were randomly selected and qRT-PCR analysis was performed. Total RNA was isolated using a TRIzol kit (Invitrogen, USA). First-strand cDNA synthesis was performed using the PrimeScriptTM First Strand cDNA Synthesis Kit (Takara, Japan). The *actin* gene (GenBank no. X63603) was used as the internal control. Primer sets were designed using Primer Premier 6.0 software^43^ (Supplementary Table S10). qRT-PCR was performed usinga SYBR Green qPCR kit (New England Biolab) according to the manufacturer’s instructions. All qRT-PCR experiments were performed in triplicate using independent samples. Expression quantification and data analysis were performed using the 2^−ΔΔCt^ method^44^.

### Data availability

The RNA-Seq raw data were deposited in the NCBI Sequence Read Archive (SRA) with the accession number SRP074868.

## Electronic supplementary material

Supplementary Information

Supplementary Table S1

Supplementary Table S2

Supplementary Table S3

Supplementary Table S4

Supplementary Table S5

Supplementary Table S6

Supplementary Table S7

Supplementary Table S8

Supplementary Table S9

Supplementary Table S10
