# Supplementary material for: Transcriptomic profile of tobacco in response to Phytophthora nicotianae infection
Source: Sci Rep. 2017 Mar 24;7:401. doi: 10.1038/s41598-017-00481-5 (PMC5428407; doi:10.1038/s41598-017-00481-5)
Supplement: Supplementary file 1 — Supplementary Information [file 41598_2017_481_MOESM1_ESM.pdf]

Transcriptomic profile of tobacco in response to *Phytophthoranicotianae* infection

Jian-Kang Yang, Zhi-Jun Tong, Dun-Huang Fang, Xue-Jun Chen, Ke-Qin Zhang, Bing-Guang Xiao

Supplementary Table S1. All genes and their gene expression data. FPKM was used to represent the expression level.

Supplementary Table S2. All genes and their expression fold change (FC) compared to controls. LogFC means of log-transformed value.

Supplementary Table S3. Annotation of 15 upregulated differentially expressed genes shared by two varieties. Annotation was from non-redundant (NR) protein database in NCBI.

Supplementary Table S4. Annotation of five unique upregulated differentially expressed genes for RBST. Annotation was from non-redundant (NR) protein database in NCBI.

Supplementary Table S5. GO enrichment for unique upregulated differentially expressed genes in HD at 12 h. *P* value was corrected with FDR.

Supplementary Table S6. GO enrichment for unique upregulated differentially expressed genes in HD at 72 h. *P* value was corrected with FDR.

Supplementary Table S7. GO enrichment for unique upregulated differentially expressed genes in RBST at 12 h and 72 h. *P* value was corrected with FDR.

Supplementary Table S8. GO enrichment for upregulated differentially expressed genes shared by HD and RBST. *P* value was corrected with FDR.

Supplementary Table S9. GO enrichment for downregulated differentially expressed genes shared by HD and RBST. *P* value was corrected with FDR.

Supplementary Table S10. Primer sequences of ten gens for qRT-PCR validation.

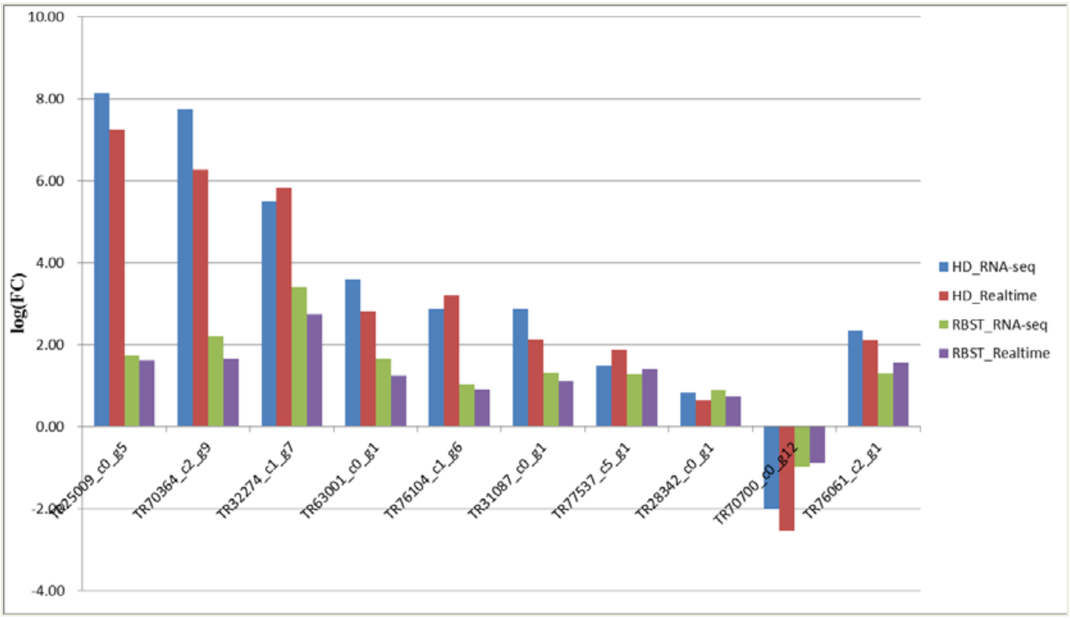

Supplementary Figure S1. qRT-PCR validation of differentially expressed ten genes. Expression profiles of selected genes as determined by qRT-PCR and DEG. The signal intensity of each transcript was normalized using *actin* gene. The y-axis shows the normalized expression level of the transcript.

Supplementary Materials and Methods

Development of the tobacco breeding line RBST

Flue-cured tobacco cultivar Coker 371-Gold possesses a dominant gene, *Ph* derived from *N. plumbaginifolia*, which confers high resistance to black shank disease, caused by race 0 of the pathogen *P. nicotianae*[S1]. Flue-cured tobacco cultivar Coker 176 possesses a dominant gene, *N* derived from N. *glutinosa*, which confers high resistance to TMV (Tobacco mosaic virus)[S2]. Coker 371-Gold is hybridized with Coker 176 to produce F1. Haploid plants were produced by crossing F1 (Coker 371-Gold × Coker 176) plant to *N. africana* (pollinator) using the maternal haploid method[S3]. Haploid plants were chromosome doubled using the midvein culture method[S4] to produce the flue-cured tobacco doubled haploid (DH) breeding line RBST, which is high resistance to both black shank disease and TMV.

Development of the flue-cured tobacco cultivar Honghuadajinyuan (HD)

The flue-cured tobacco cultivar HD was developed from a spontaneous mutant with deep red flower in a Yunnan farmer's field growing the flue-cured tobacco cultivar Gold Dollar, which is the farmer variety in USA[S5]. HD could produce high quality tobacco leaves, but it was susceptible

to black shank disease.

Supplementary References

S1. Johnson, E. S., Wolff, M. F., Wernsman, E. A., Atchley, W. R. & Shew, H. D. Origin of the black shank resistance gene, Ph, in tobacco cultivar Coker 371-Gold. *Plant Dis* **86**, 1080–1084 (2002).

S2. Lewis, R. S., Milla, S. R. & Levin, J. S. Molecular and genetic characterization of Nicotiana glutinosa L. chromosome segments in Tobacco mosaic virus-resistant tobacco accessions. *Crop Sci* **45**, 2355–2362 (2005).

S3. Burk, L. G., Gerstel, D. U. & Wernsman, E. A. Maternal haploids of Nicotiana tabacum L. from seed. *Science* **206**, 585 (1979).

S4. Kasperbauer, M. A. & Collins, G. B. Reconstitution of diploids from leaf tissue of anther derived haploids in tobacco. *Crop Sci* **12**, 98–101 (1972).

S5. Moon, H.S. *et al*. Changes in Genetic Diversity of U.S. Flue-Cured Tobacco Germplasm over Seven Decades of Cultivar Development. *Crop Sci* **49**, 498–508 (2009).
